# Prevention of Peritendinous Adhesions Using an Electrospun DegraPol Polymer Tube: A Histological, Ultrasonographic, and Biomechanical Study in Rabbits

**DOI:** 10.1155/2014/656240

**Published:** 2014-07-02

**Authors:** Gabriella Meier Bürgisser, Maurizio Calcagni, Angela Müller, Eliana Bonavoglia, Gion Fessel, Jess G. Snedeker, Pietro Giovanoli, Johanna Buschmann

**Affiliations:** ^1^Division of Plastic Surgery and Hand Surgery, University Hospital Zurich, ZKF, Sternwartstrasse 14, 8091 Zurich, Switzerland; ^2^ab medica, Via Nerviano 31, 20020 Lainate, Milan, Italy; ^3^Department of Orthopedics, Uniklinik Balgrist, Forchstrasse 340, 8008 Zurich, Switzerland

## Abstract

*Purpose*. One of the great challenges in surgical tendon rupture repair is to minimize peritendinous adhesions. In order to reduce adhesion formation, a physical barrier was applied to a sutured rabbit Achilles tendon, with two different immobilization protocols used postoperatively. *Methods*. Thirty New Zealand white rabbits received a laceration on the Achilles tendon, sutured with a 4-strand Becker suture, and half of the rabbits got a DegraPol tube at the repair site. While fifteen rabbits had their treated hind leg in a 180° stretched position during 6 weeks (adhesion provoking immobilization), the other fifteen rabbits were recasted with a 150° position after 3 weeks (adhesion inhibiting immobilization). Adhesion extent was analysed macroscopically, via ultrasound and histology. Inflammation was determined histologically. Biomechanical properties were analysed. *Results*. Application of a DegraPol tube reduced adhesion formation by approximately 20%—independently of the immobilization protocol. Biomechanical properties of extracted specimen were not affected by the tube application. There was no serious inflammatory reaction towards the implant material. *Conclusions*. Implantation of a DegraPol tube tightly set around a sutured tendon acts as a beneficial physical barrier and prevents adhesion formation significantly—without affecting the tendon healing process.

## 1. Introduction

Tendon repair is a field in surgery where improvements are still welcome. Complications such as scar formation and adhesion in the tendon sheath in the early healing phase up to 6 weeks do occur in 7 to 15% of the cases, which leads to increased work disability and costs [1]. In particular, there is a need to reduce peritendinous adhesion formation because this is reported to be the most common complication following flexor tendon repair in the hand [2]—but also in the Achilles tendons, functional complications caused by adhesion are reported [3]. Hence, different antiadhesion strategies to improve the gliding capability of the regenerating tendon are being developed [4]. First of all, the postoperative treatment has to be optimized with respect to its impact on the adhesion extent as it is well known that for example in Achilles tendon regeneration the ankle angle plays a crucial role [5]. Secondly, physical antiadhesion barriers consisting of biomaterials such as fibronectin [6], collagen [3], or silk [7] have been reported to show positive effects with respect to reducing adhesion formation. Finally, antiadhesive agents were beneficially administered such as 5-fluorouracil [8], hyaluronic acid, and ADCON-T/N, being a gelatine-polyglycan ester compound [9] or anti-inflammatory drugs like ibuprofen [10].

Hence, concepts of adhesion prevention include postoperative treatment strategies and pharmacological as well as mechanical agents. An effective mechanical barrier should be biocompatible, biodegradable to achieve tissue integration and prevent buldging. Furthermore, it should be easy in surgical handling during implantation and cause only a low inflammatory response. The polymer DegraPol which is a copolymer of poly-hydroxy-butyrate and *ε*-caprolactone meets all these requirements [11]. It has been shown to be an ideal scaffold material for tenocytes [12]. In recent studies, we checked the cellular response toward classic DegraPol in Achilles tendon repair in a rabbit model (mimicking the biomechanical properties of human flexor tendons of the hand) and found no adversary effects [13]. Subsequently, we synthesized a new more elastic DegraPol polymer in order to facilitate surgical handling [14].

In the study presented here, an electrospun DegraPol tube as an antiadhesive was investigated. Therefore, the adhesion extent of lacerated rabbit Achilles tendons 6 weeks after surgery either with or without the application of a DegraPol tube was determined by macroscopic evaluation, by dynamic ultrasound, and by histology. The rabbit Achilles tendon is surrounded by a leaflet of fascia and it has similar biomechanical properties as the human flexor tendons of the hand [15, 16]. In order to modulate the adhesion in the rabbit Achilles tendon model, we used two different postoperative immobilization protocols: the ankle angle of the cast was changed after 3 weeks from 180° to 150° (adhesion inhibiting immobilization protocol) or the cast had an ankle angle of 180° for the full 6-week period (adhesion provoking immobilization protocol). Biomechanical properties including load until failure, cross sectional area (CSA), stiffness, E-modulus, and failure stress were determined. Additionally, inflammatory reaction was determined based on macrophages and lymphocytes. Hence, the two hypotheses were that (i) the application of a DegraPol tube around the repaired tendon would reduce adhesion formation without an inflammatory reaction in both postoperative treatment models and (ii) the biomechanical properties of the regenerated tendons would not be adversely affected by the implantation of a DegraPol tube.

## 2. Materials and Methods

### 2.1. Block Copolymer DegraPol

A biodegradable polyester urethane polymer (trade name DegraPol) with poly-hydroxy-butyrate as a crystalline segment and *ε*-caprolactone as a soft segment was synthesized according to an adapted protocol [14]. Briefly, for the synthesis of the block copolymer, 25 wt% of poly(3-(R)-hydroxybutyrate)-co-(*ε*-caprolactone)-diol (*M*_*n*_ = 2824 g mol^−1^) and 75 wt% with *M*_*n*_ = 1000 g mol^−1^ poly(*ε*-caprolactone)-diol-co-glycolide (15 mol% glycolide 85 mol% *ε*-caprolactone) were dissolved in 1.4-dioxane and dried by heating and refluxing the solvent over molecular sieves (pore size 0.4 nm). The reaction mixture was cooled to 83°C before the stoichiometric amount, with respect to the two diols, of 2,2,4-trimethylhexane-diisocyanate (TMDI) was added. After about one day of reaction, three portions of dibutyltin dilaurate (20 ppm) were added within 1 day in order to reach molecular weight of 110 kDa. The polymer was precipitated in dry ice cooled hexane isomers and subsequently purified via dissolution in chloroform and filtration over a silicagel 60 (Fluka) column. A second precipitation in cooled ethanol ended the process.

### 2.2. Scaffold Fabrication and Characteristics

**Figure 1:**
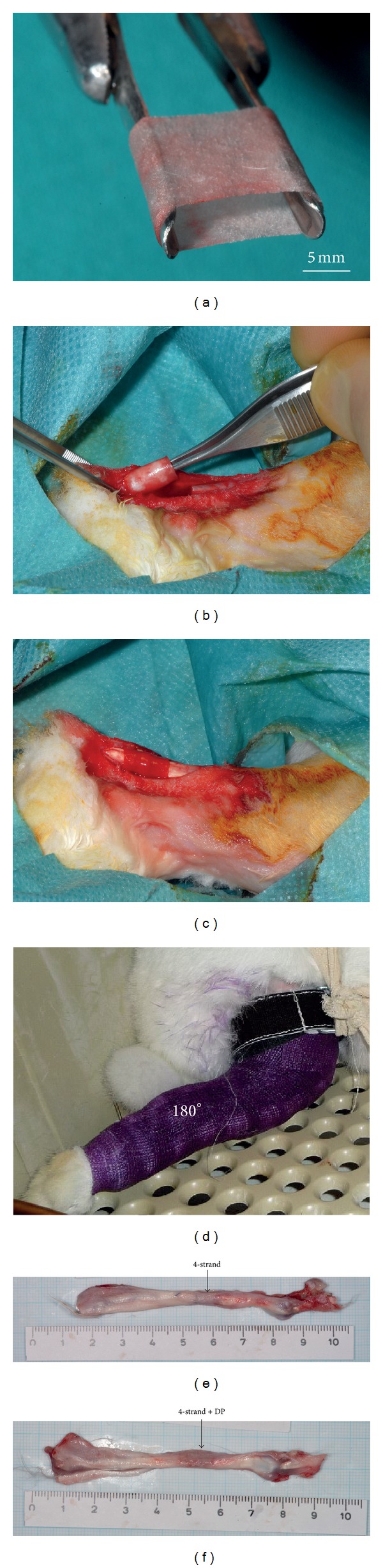
(a) Very elastic DegraPol tube used for mechanical blockage of regenerating tendon tissue and surrounding tissue, (b) and (c) implantation of tube in addition to a conventional 4-strand Becker suture, (d) New Zealand white rabbit with a cast having an ankle angle of 180° (fully stretched), (e) extracted tendon specimen with 4-strand suture and (f) extracted tendon specimen with DP tube application in addition to 4-strand suture.

The electrospinning setup was assembled in-house and consisted of a syringe pump (Racel Scientific Instruments Inc., USA), a spinning head consisting of a central stainless steel tube (1 mm inner diameter and 0.3 mm wall thickness, Angst & Pfister AG, Switzerland), a cylindrical rotating aluminum mandrel for fiber collection (length: 100 mm, diameter: 4 mm), and a DC high voltage supply (Glassman High Voltage Inc., USA). A 25 wt% solution of the DegraPol (DP) in chloroform (Fluka, puriss., Switzerland) was prepared by dissolving the polymer under stirring overnight. Electrospinning voltage (15 kV) was applied with a high voltage supply between a needle and the rotating cylindrical collector (20 cm apart from each other). As-spun tubes (with randomly oriented fibres) were removed from the target by slightly swelling them with ethanol (Fluka, puriss., Switzerland) and then dried under vacuum at room temperature. In Figure 1(a), the high elasticity of such a tube is shown. The overall mesh porosity *P* was calculated according to *P* = (1 − *ρ*/*ρ*_0_) × 100 (%), where *ρ* denotes the density of the electrospun mesh and *ρ*_0_ is the bulk density of the electrospun mesh determined gravimetrically using weights of precisely cut samples of defined area and thickness; *P* was 75.2 ± 0.4%. The wall thickness was 357.0 ± 17.7 *μ*m as determined by means of scanning electron microscopy (SEM, FEI, Nova NanoSEM 450). The fibres of the electrospun tube were randomly oriented. The tubes had an inner diameter of 4 mm and were cut into pieces of 1 cm in length. Their degradation half-lives were determined to be approximately 3 months.

### 2.3. Animals

For the* in vivo* study, thirty female New Zealand white rabbits aged 12 to 16 weeks were used (Charles River, Research Models and Services, Germany). They were specific pathogen free (SPF). All animals were housed in pairs in two interconnected cages, each of them with a bottom area of 70 cm × 70 cm and a height of 62 cm (Indulab, Switzerland). The animals were maintained under controlled conditions: temperature 22 ± 1°C, 45% relative humidity, 15 air changes per hour, and a light/dark rhythm of 12 hours. The rabbits had free access to water (automatic water supply), autoclaved hay, and straw* ad libitum* as well as to standard pellet diet (Kliba Nafag, Nr. 3410, Provimi Kliba AG, Switzerland). Ethical approval was obtained for the experiments from the veterinary office of Zurich, Switzerland (reference numbers 92/2009 and 193/2012). Prior to surgery, all animals were acclimatized to their environment for two weeks.

### 2.4. Achilles Tendon Repair

Before implantation, the DP tubes were sterilized with ethylene oxide at 38°C. The rabbits received premedication with 65 mg/kg body weight Ketamine and 4 mg/kg Xylazine. A venous catheter was inserted in the marginal ear vein. The rabbits were intubated with Propofol i.v. 0.6 mg/kg–1.3 mg/kg. Anaesthesia was maintained with 1-2% isofluorane. In order to ensure systemic analgesia during the time of operation, 0.2-0.3 mg/kg body weight Butorphanol (Dr. E. Graeub AG, Berne, Switzerland) was applied preoperatively. The hind legs were cleaned with iodine. The Achilles tendon exposure was obtained through a paratendineal incision of cutis, subcutis, and fascia. The medial and lateral* M. gastrocnemius* of the Achilles tendon complex was then sliced perpendicularly to the length of the tendon 2.0 cm above the* calcaneus* and the two tendon stumps were sutured (4-strand Becker suture) using a USP 4.0 polypropylene thread. In case of DP tube application, one of the two tendon stumps was pulled through the DP tube before suturing (Figures 1(b) and 1(c)). Subsequently the wound was closed with a running suture (using a USP 6.0 polypropylene fiber) of the fascia and interrupted skin. Immediately after surgery, a Duragesic matrix patch (Janssen-Cilag AG, Switzerland) was applied with 4.2 mg Fentanyl per patch in order to provide analgesia for about 72 hours with 25 *μ*g/h Fentanyl. Postoperative treatment included a cast having an angle of 180° at the ankle for a total of 12 rabbits (6 with and 6 without tube; adhesion provoking immobilization) (Figure 1(d)). The cast was well padded and it was changed after 3 weeks. For the other 12 rabbits, the cast was also changed after 3 weeks; however, a cast with a smaller angle of 150° was applied (adhesion inhibiting immobilization). Great attention was paid to make the casts not too tight so that it was tolerated well by the rabbits (they did not bite the cast). Six weeks after surgery, the rabbits were euthanized in deep anaesthesia (100 mg/kg Ketamine and 4 mg/kg Xylazine) with 80 mg/kg Pentobarbital (Esconarkon* ad us. vet*., Switzerland).

### 2.5. Treatment Groups

**Figure 2:**
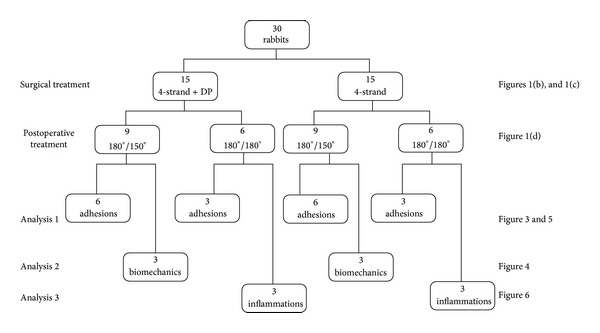
Experimental design depicting number of rabbits used for the corresponding surgical and postoperative treatments. Accordingly, the three different analysis approaches are given (adhesion = determination of adhesion extent by macroscopic, dynamic ultrasound and histological analyses; biomechanics = determination of load until failure, cross-sectional area, stiffness, failure stress and elastic modulus; inflammation = determination of macrophage and lymphocyte density). Moreover, links to figure numbers of the respective results are given.

The thirty rabbits were randomly distributed into eight groups with *n* = 3 or 6 (Figure 2). All were operated on one hind leg [17]. The tendons of twenty-four rabbits were analysed histologically (eighteen of them for adhesion scoring (twelve in the 180°/150° and six in the 180°/180° group), six for inflammation analysis, all in the 180°/180° group), with groups classified by the application of a DP tube in addition to a 4-strand Becker suture (application or no application) and by the casting protocol (ankle angle change from 180° to 150° (180°/150° group) or 180° for the full 6-week period with a cast change after 3 weeks (180°/180° group)). The tendons of six rabbits were analysed biomechanically, all receiving a 180°/150° immobilization, including three animals with DP and three without. The counter legs that were not treated (NT) served as control.

### 2.6. Rating during Tendon Extraction

Achilles tendons including* flexor digitorum superficialis* and surrounding tissue were extracted. During extraction, the extent of adhesion formation was macroscopically evaluated (carefully paying attention to not move the tendon in the sheath) and scored semiquantitatively with 0 = good gliding (no adhesion), 1 = middle gliding (some adhesion) and 2 = no gliding (maximum adhesion). The surgeon was blinded to the treatment.

### 2.7. Dynamic Ultrasound

The Achilles tendons of all animals were imaged with high-frequency ultrasound before surgery and at the end point of the experiments before explanting the tendons. Ultrasound imaging was performed with an ultrasound unit (iU22 Ultrasound System, Philips Healthcare, Switzerland) with a linear high-frequency hockey-stick probe of 17.5 MHz (L17-5io Broadband Compact Linear Array Transducer, Philips Healthcare, Switzerland). The examination protocol consisted of a dynamic imaging of the gliding Achilles tendon by moving the* flexor digitorum superficialis* of the rabbit paw. All measurements were done freehand. The films of the gliding tendons were scored with 0 = good gliding (no adhesion), 1 = middle gliding (some adhesion) and 2 = no gliding (maximum adhesion) (see Supplementary Videos 1 and 2 (see Supplementary Material available online at http://dx.doi.org/10.1155/2014/656240)). Colour gain adjustment was calibrated on the counter leg that was not treated (healthy side).

### 2.8. Quantification of Adhesion Extent and Inflammation by Histology

After extraction, the Achilles tendon specimens were immediately frozen at −20°C. After being thawed to room temperature, they were dehydrated, paraffin-embedded, and sectioned into 5 *μ*m thick slices, which were cross sections in the DP tube region (perpendicular to the Achilles tendon). After being deparaffinized with xylene and rehydrating the sections (descending gradient of ethanol), they were differently stained: Picrosirius red and H&E according to commonly established procedures.

Picrosirius red stained sections were used to quantify the adhesion extent at 8x magnification (Leica EZ4D microscope, Switzerland). Here, adhesion formation was quantified in five subsequent cross sections separated by 2.0 mm using a method by Tan et al. [18]. Analysis was done in a blinded fashion. The percentage of adhesion was calculated by the length of the contact region of the tendon under view with the surrounding tissue divided by the total perimeter. The length of the contact region and the whole perimeter were determined using* synedra View 3* software (version 3. 1. 0. 3.).

Inflammation zones based on macrophages and on lymphocytes were evaluated in the H&E-stained sections at 100x and 200x magnification based on their morphology (5 FOV of each object, semiquantitative analysis); scores: 1 = healthy tendon tissue, 2 = some fine films of inflammation, 3 = more fine films of inflammation, 4 = few zones (areas) of inflammation, and 5 = many zones (areas) of inflammation.

### 2.9. Biomechanical Tests

Before measurement at room temperature (21°C), the tendons were thawed overnight at 4°C. All tendons were harvested from the hind legs including the muscle and the* calcaneus*. On the muscle side, the samples were mounted in serrated clamps after being wrapped in two pieces of cloth to reduce slippage [19], and on the bone side, a device fixing the* calcaneus* in a rectangular position to the tendon was used. All samples were tested in uniaxial tension to failure at 1 mm min^−1^ speed on a Zwick 1456 tensile testing machine (1 kN load-cell, 0.1 *μ*m extensiometer, TestXpert 10, Germany) with preconditioning (10 cycles to 10 N). The samples were sprayed with phosphate buffered saline during measurement in order to prevent drying. Load until failure (N) was determined as the maximum load measured.

The CSA was determined 2.0 cm above the* calcaneus* by a custom designed linear laser scanner adapted by Vergari et al. with *n* = 6 per specimen before tensile testing [20, 21]. The load until failure (N) was divided by the thus-determined CSA at the repair site (m^2^) resulting in the failure stress at the repair site (Pa). The elastic modulus (E-Modulus; Pa) was determined in the stress-strain curves. Moreover, the stiffness (N/mm) was determined in the force-elongation curves.

### 2.10. Statistical Analysis

Histomorphometric and biomechanical data were analysed with StatView 5.0.1. One-way analysis of variance (one-way ANOVA) was conducted. Pairwise comparison probabilities (*P*) were calculated using Fisher's PLSD. *P* values <0.05 were considered significant. Values were expressed as means ± standard deviations.

## 3. Results

### 3.1. Adhesion Provoking and Adhesion Inhibiting Immobilization

The two different postoperative casting protocols had a significant impact on the adhesion extent. A change of the ankle angle from 180° to 150° after 3 weeks reduced the adhesion extent significantly from 82.7 ± 9.7% to 31.9 ± 9.8% (*P* < 0.05) when the tendon laceration was repaired by a conventional 4-strand Becker suture (Supplementary Table 1). Accordingly, in the tube-treated group, the change of the ankle angle after 3 weeks leads to a reduction in the adhesion extent from 69.3 ± 9.8% to 15.6 ± 10.9% (*P* < 0.05).

### 3.2. Impact of DegraPol Tube on Adhesion

**Figure 3:**
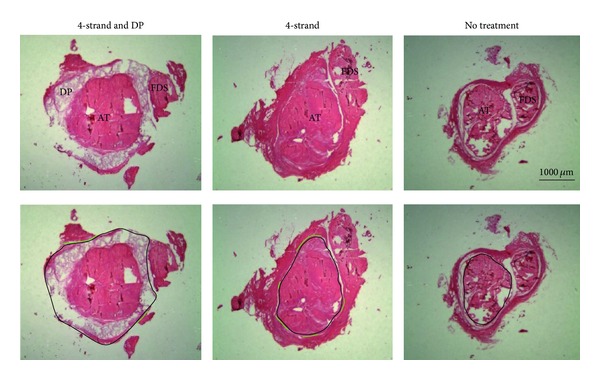
Picrosirius red stained 5 *μ*m histological sections of the three groups considered at 8x magnification.* Key*: 4-strand + DP = 4-strand Becker suture and application of DegraPol tube; 4-strand = 4-strand Becker suture; no treatment = control group without any intervention; DP = DegraPol, AT = Achilles tendon; FDS =* Flexor digitorum superficialis*. The black lines in the lower figures denote the whole circumference, while the green lines depict the adhesion formation.

The extent of adhesion formation was independently evaluated by two different persons using a semiquantitative scoring system by macroscopic observation during extraction (MC) and by the percentage of contact regions of the Achilles tendon to its surrounding tissue calculated from five subsequent histological cross-sections (GMB) (Figure 3). There was a statistically significant difference in adhesion formation between tube-treated specimen and specimen without tube. The implantation of a DP tube reduced the contact area to the surrounding tissue significantly from 31.9 ± 9.8% to 15.6 ± 10.9% in adhesion inhibiting postoperative model (*P* < 0.05), while it reduced the contact area from 82.7 ± 9.7% to 69.3 ± 9.8% in the adhesion provoking postoperative model (*P* < 0.05).

### 3.3. Biomechanics

**Figure 4:**
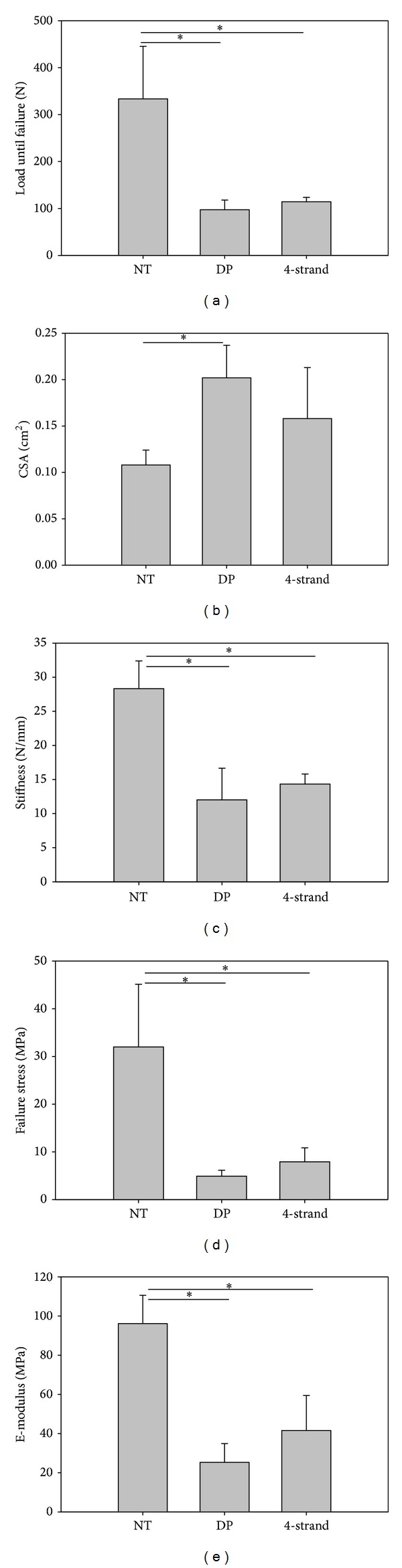
Load until failure (a), CSA (b), stiffness (c), failure stress (d), and E-modulus (e) determined for the tendons of rabbits with a 180°/150° casting regime.* Key*: NT = no treatment (contralateral legs); DP = 4-strand Becker suture and a DegraPol tube; 4-strand = 4-strand Becker suture. *P* values <0.05 are marked by asterisk (^∗^). Sample size was *n* = 3 for DP and 4-strand groups, respectively, and *n* = 6 for NT group.

Figure 4 shows the biomechanical results for rabbits with the 180°/150° casting regime (adhesion inhibiting model). There were no statistical significant differences between the DP treated and the mere 4-strand treated specimen (neither for load until failure (*P* = 0.81) nor for CSA (*P* = 0.13), for stiffness (0.44), for E-Modulus (0.20), and for failure stress (0.72)), indicating no adverse effect of the polymer tube on biomechanical properties of the healing tendon.

### 3.4. Correlation of Macroscopic and Histological Evaluation

**Figure 5:**
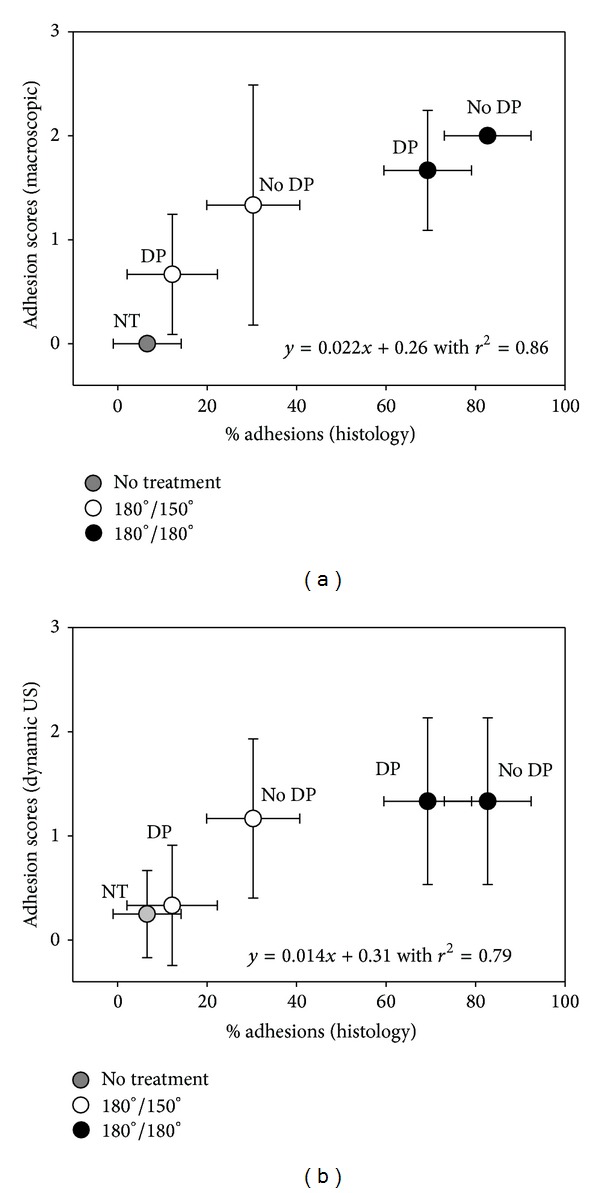
Adhesion scores determined by semiquantitative scoring on observation during tendons extractions (a) and by dynamic ultrasound (b) correlated well with the percentage of adhesions to the surrounding tissue (*r* ^2^ = 0.86 and 0.79, resp.). Pairwise comparison probabilities (*P*) were calculated using Fisher's PLSD and the Bonferroni post hoc test. All *P* values are given in Supplementary Table 1.* Key:* DP = DegraPol tube application; no DP = mere 4-strand suture; NT = no treatment, control; empty circles = immobilization protocol where the ankle angle of the cast was changed from 180° to 150° after 3 weeks; black circles = immobilization protocol where the ankle angle of the cast was 180° during 6 weeks (cast renewed after 3 weeks). Sample size was *n* = 3 for 180°/180°, *n* = 6 for 180°/150° group, and *n* = 18 for NT group.

Semiquantitative adhesion scores gathered immediately after tendon extraction were correlated to the quantitative analysis of adhesion determined in histological sections (Figure 5(a)). A positive correlation was found (*r* ^2^ = 0.86).

### 3.5. Correlation of Dynamic Ultrasound and Histological Evaluation

Semiquantitative adhesion scores from dynamic ultrasound were correlated with the quantitative analysis of adhesion determined in histological sections (Figure 5(b)). A positive correlation was found (*r* ^2^ = 0.79).

### 3.6. Inflammation

**Figure 6:**
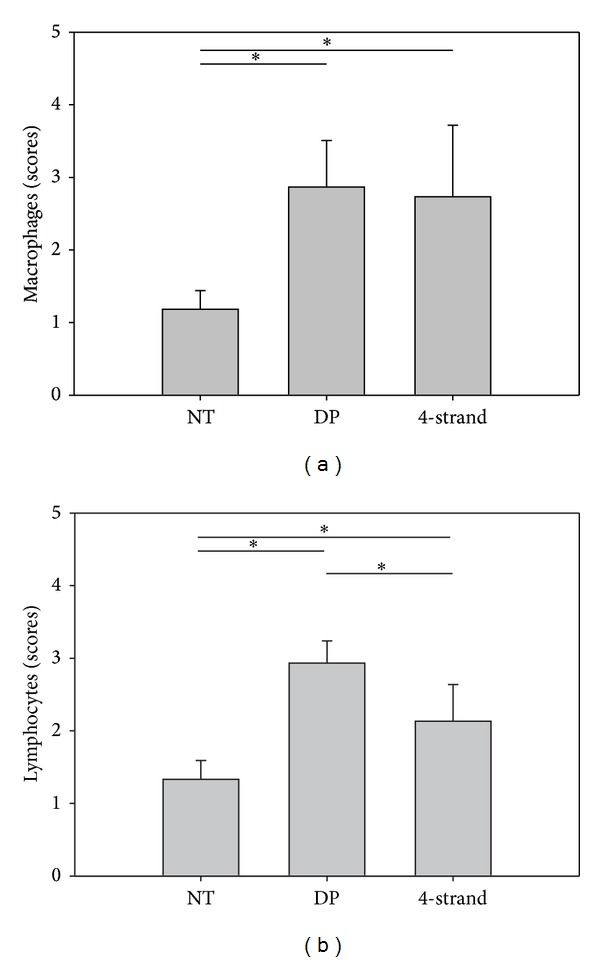
Semiquantitative evaluations of macrophages (a) and lymphocytes (b) in the tendon tissue. Scores: 1 = healthy tendon tissue, 2 = some fine films of inflammation, 3 = more fine films of inflammation, 4 = few zones (areas) of inflammation, 5 = many zones (areas) of inflammation. Sample size was *n* = 3 for all groups; except for NT group (*n* = 6).

Inflammation was semiquantitatively scored and found to be not significantly different whether a DP tube was implanted or not for macrophages (Figure 6(a)); only lymphocyte analysis showed a significant higher cell density for the DP treated specimen (*P* = 0.05) (Figure 6(b)).

## 4. Discussion

The most common problem arising during tendon healing is the formation of fibrous adhesions to the surrounding tissue [2]. The etiology is based on the destruction of a cell-retentive layer (basement membrane) on the tendon surface. At the site of the tendon rupture, cells exit from the damaged tendons where the basement membrane is damaged [22]. Thus, adhesions are mostly evoked by scarring between two damaged surfaces [23]. Many studies dealing with the application of lubricants have been published [18, 24–26]—the outcome being controversial. For example, while some authors claim a beneficial effect of hyaluronic acid (based on adhesion scoring and cell density) [27], others found adhesion to be unaffected [28]. Physical barriers such as collagen membranes [3], PGA membranes [29], PLGA membranes [10], or spontaneously forming hydrogels [30] positively affected adhesion formation, although their use in the clinical set up is still very scarce due to limited tissue integration, inflammatory reactions, cell stress caused by lowered pH, or decreasing reduced final strength of the regenerated tendon.

Different rehabilitation protocols are reported to have an impact on the clinical outcome of tendon rupture repairs. Early active motion protocols go along with lower complication rates [31]. In an experimental study by Pihlajamäki et al., the cellular composition at the repair site 6 weeks after surgery of the healing rabbit Achilles tendon was similar whether a cast was applied or not [29]. In contrast, other studies denote the importance of the specific postoperative casting regime [32]. Besides the length of the application period of the cast, the ankle angle is one important parameter [5].

With the intention to drastically modulate the adhesion extent, we used two different postoperative immobilization protocols. The ankle angle of the cast was either changed or not 3 weeks after surgery. Early active or passive motion was not included—in contrast to other studies [33, 34], because a defined and standardized early motion protocol is very difficult to realize in rabbits. Therefore, a cast was applied for a total time period of 6 weeks (end of experiments). While one group received the whole 6 weeks a 180° cast (renewed after 3 weeks, provoking adhesion formation), the other group had a 180° cast for the first 3-week period and then a new 150° cast for the second 3-week period (inhibiting adhesion formation). A totally stretched position was chosen because the Achilles tendon is perfectly relaxed and the rabbit's foot is in a position which is usually taken during rest. The adhesion inhibiting protocol including a change in the ankle angle led to a clear reduction of adhesion by over 50%, not only because some initial fibrous adhesion were decreased by the intervention of a positional change among the involved tissue layers, but also by having the angle a little bit closer to angles occurring during jumping [35].

Mechanical blockage by an electrospun DegraPol polymer tube 6 weeks after surgery was tested in both postoperative treatment models and the adhesion provoking as well as the adhesion inhibiting model. We had tested DegraPol before with respect to its cell response* in vivo* 12 weeks after operation [13]. The polymer DegraPol is a biocompatible and totally biodegradable block copolymer. With its hard and soft segments that can be varied user-defined in weight ratios during production, it allows a wide range of degradation half-lives, stabilities, and biomechanical characteristics. Originally, it was developed as a scaffold material for bone reconstruction [36]. However, DegraPol was also shown to be a good scaffold for tenocytes [37]. Even after having changed its synthesis protocol in order to make the polymer more elastic to facilitate the surgeon's pulling it over the repair site, no adverse cellular effects 12 weeks after operation were observed upon implantation [14]. The very high elasticity of the electrospun DegraPol mesh denoted by an elongation at break of 544 ± 68% is a big advantage over other mechanical barrier options—even the best ones with respect to this property—such as electrospun PLGA having an elongation at break of “only” 324 ± 78% [38]. Moreover, degrading DegraPol is pH-neutral while other polymers used as implant materials degrade with lowering the pH (like PLGA).

In this study, the quantitative determination of the adhesion extent by histological analysis clearly showed that the implantation of a DegraPol tube around the conventionally sutured tendon reduced the peritendinous adhesion significantly by around 20%—independent of the postoperative treatment mode. Moreover, this antiadhesive effect was clearly supported by our semiquantitative findings based on macroscopic inspection and evaluation and scoring of the gliding capacity by dynamic ultrasound, both correlating positively to the histological findings. Although other studies report much higher reduction of adhesion extent up to 88% by using, for example, an Fe^3+^ crosslinked carboxy-methylcellulose barrier, such results are not directly comparable to our findings because the adhesion extent was determined at a different time point—already after 2 weeks [39], when peritendinous adhesion formation is not yet completed [40]. Importantly, the implantation of our electrospun DP tube did not lead to a decrease in failure strength nor in other biomechanical parameters such failure stress, tensile modulus, or stiffness—standing in contrast to other studies using, for example, cortisone as antiadhesive that leads to great problems caused by decreased biomechanical properties after implantation [41].

Though not statistically significantly different, the failure stress and the E-Modulus—two typical material properties [42]—of the DP tube specimen were approximately 30% lower compared to no-tube specimen; this is mainly caused by the approximately 30% larger CSA provoked by the DP tube that is not yet degraded fully after 6 weeks. In addition, inflammatory reaction to the implant material was only found to be marginally increased for lymphocytes, but not for macrophages which was the abundant subpopulation of inflammatory cells, and which might cause problems when materials that are less biocompatible such as cellulose [43] are used as physical barriers.

Although adhesion formation was analyzed by macroscopic evaluation, dynamic ultrasound, and quantitative histology, it might be favorable in future studies to quantify the gliding capability of the rabbit Achilles tendon by range-of-motion analysis [44]. Moreover, inflammatory reactions towards the implant material might be analyzed in more details using immunohistochemistry methods [45].

## 5. Conclusion

In summary, we could demonstrate that an electrospun DegraPol tube acts as an efficient physical barrier enabling a reduction of peritendinous adhesion without changing biomechanical characteristics and not affecting inflammation—and regardless of the postoperative treatment protocol. In both treatment models, we found a significant adhesion reduction of approximately 20%. The beneficial impact of this electrospun material could be clearly demonstrated for this purpose. Thus, peritendinous adhesion prevention by a DegraPol tube is a promising approach and may find its way to clinical application after some further modifications will be undertaken; therefore, we plan to load such a tube with tendon growth factors inside in order to accelerate tendon healing, thus generating a device that is physically blocking adhesion formation outside, while simultaneously promoting the healing process inside.

## Supplementary Material

Supporting Information SI Table 1: P-Values of ANOVA for gliding scores determined macroscopically during extraction (denoted *Macroscopic*), dynamic ultrasound (denoted Ultrasound) and percentage of contact area to the surrounding tissue determined by analysis of histological sections (denoted *Histological*); once with the Fisher's PLSD (abbreviation PLSD) post hoc test (p < 0.05 is significant) and for comparison with the Bonferroni adjustment (BF) where comparisons are not significant unless the corresponding *p*-value is less than 0.005 (∗ = significance).
Supporting Information SI Video 1: For dynamic ultrasound, a semi-quantitative scoring system was used (see materials and methods section). In video 1, a typical video is shown for a good gliding of the rabbit Achilles tendon (score 0), having no adhesion.
Supporting Information SI Video 2: For dynamic ultrasound, a semi-quantitative scoring system was used (see materials and methods section). In video 2, a typical video is shown for a strong adhesion of the Achilles tendon towards the surrounding tissue (score 2).
Supporting Information SI Table 1: P-Values of ANOVA for gliding scores determined macroscopically during extraction (denoted *Macroscopic*), dynamic ultrasound (denoted Ultrasound) and percentage of contact area to the surrounding tissue determined by analysis of histological sections (denoted *Histological*); once with the Fisher's PLSD (abbreviation PLSD) post hoc test (p < 0.05 is significant) and for comparison with the Bonferroni adjustment (BF) where comparisons are not significant unless the corresponding *p*-value is less than 0.005 (∗ = significance).
