# Supplementary material for: Prevention of Peritendinous Adhesions Using an Electrospun DegraPol Polymer Tube: A Histological, Ultrasonographic, and Biomechanical Study in Rabbits
Source: Biomed Res Int. 2014 Jul 2;2014:656240. doi: 10.1155/2014/656240 (PMC4101979; doi:10.1155/2014/656240)
Supplement: Supplementary file 1 — Supporting Information SI Table 1: P-Values of ANOVA for gliding scores determined macroscopically during extraction (denoted Macroscopic), dynamic ultrasound (denoted Ultrasound) and percentage of contact area to the surrounding tissue determined by analysis of histological sections (denoted Histological); once with the Fisher's PLSD (abbreviation PLSD) post hoc test (p < 0.05 is significant) and for comparison with the Bonferroni adjustment (BF) where comparisons are not significant unless the corresponding p-value is less than 0.005 (∗ = significance). Supporting Information SI Video 1: For dynamic ultrasound, a semi-quantitative scoring system was used (see materials and methods section). In video 1, a typical video is shown for a good gliding of the rabbit Achilles tendon (score 0), having no adhesion. Supporting Information SI Video 2: For dynamic ultrasound, a semi-quantitative scoring system was used (see materials and methods section). In video 2, a typical video is shown for a strong adhesion of the Achilles tendon towards the surrounding tissue (score 2). Supporting Information SI Table 1: P-Values of ANOVA for gliding scores determined macroscopically during extraction (denoted Macroscopic), dynamic ultrasound (denoted Ultrasound) and percentage of contact area to the surrounding tissue determined by analysis of histological sections (denoted Histological); once with the Fisher's PLSD (abbreviation PLSD) post hoc test (p < 0.05 is significant) and for comparison with the Bonferroni adjustment (BF) where comparisons are not significant unless the corresponding p-value is less than 0.005 (∗ = significance). [file 656240.f1.zip › supplementary table1.pdf]

Supporting Information SI Table 1 P-Values of ANOVA for gliding scores determined macroscopically during extraction (denoted *Macroscopic*), dynamic ultrasound (denoted *Ultrasound*) and percentage of contact area to the surrounding tissue determined by analysis of histological sections (denoted *Histological*); once with the Fisher's PLSD (abbreviation PLSD) post hoc test ($p < 0.05$ is significant) and for comparison with the Bonferroni adjustment (BF) where comparisons are not significant unless the corresponding *p*-value is less than 0.005 (* = significance).

Group I	*Group II*	*Macroscopic*		*Ultrasound*		*Histological*	
		PLSD	*BF*	*PLSD*	*BF*	*PLSD*	*BF*
DP, 180/150	DP, 180/180	.0152*	.0152	.1434	.1434	<.0001*	<.0001*
DP, 180/150	NT	.0364*	.0364	.8832	.8832	.2769	.2769
DP, 180/150	No DP, 180/150	.0912	.0912	.2170	.2170	<.0001*	<.0001*
DP, 180/150	No DP, 180/180	.0021*	.0021*	.1434	.1434	<.0001*	<.0001*
DP, 180/180	NT	<.0001*	<.0001*	.0733*	.0733*	<.0001*	<.0001*
DP, 180/180	No DP, 180/150	.3847	.3847	.7993	.7993	<.0001*	<.0001*
DP, 180/180	No DP, 180/180	.3847	.3847	equal	equal	.0080*	.0080
NT	No DP, 180/150	.0002*	.0002*	.1232	.1232	<.0001*	<.0001*
NT	No DP, 180/180	<.0001*	<.0001*	.0733*	.0733*	<.0001*	<.0001*
No DP, 180/150	No DP, 180/180	.0912	.0912	.7993	.7993	<.0001*	<.0001*
